# Cutaneous manifestations of *Mycobacterium gordonae *infection described for the first time in Italy: a case report

**DOI:** 10.4076/1757-1626-2-6828

**Published:** 2009-07-31

**Authors:** Caterina Foti, Vincenza Sforza, Caterina Rizzo, Giovanna De Pascale, Domenico Bonamonte, Anna Conserva, Antonio Tarantino, Camilla Stella, Stefania Cantore, Roberto Felice Grassi, Andrea Ballini, Danila De Vito, Giovanni Angelini

**Affiliations:** 1 Department of Internal Medicine, Immunology and Infectious Diseases, Unit of Dermatology, University of Bari (P.zza Giulio Cesare n.11) Bari (70124), Italy; 2 Institute "Villa del Sole" Dermatology Unit, (C.so A. De Gasperi n.413) Bari (70100), Italy; 3 Department of Pharmaco-Biology, University of Bari (Via Orabona n.4) Bari (70100), Italy; 4 Department of Dental Sciences and Surgery, University of Bari (P.zza Giulio Cesare n.11) Bari (70124), Italy

## Abstract

**Introduction:**

*Mycobacterium gordonae *is one of the least pathogenic of the mycobacteria. This pathogen may produce caseating or non-caseating granulomas, and skin lesions showing acute or chronic inflammation with scattered histiocytes and giant cells have been seen. The mortality rate is less than 0.1%. *Mycobacterium gordonae *may be a marker of severe immunosuppression in patients infected with human immunodeficiency virus.

**Case presentation:**

We report a case of *Mycobacterium gordonae *infection in an 86-year-old woman and discuss the problems inherent to the identification and treatment of this emerging pathogen. *Mycobacterium gordonae *strain we isolated was resistant to trimethoprim-sulfamethoxazole but sensitive to ciprofloxacin, and long term administration (six months) induced complete healing of the cutaneous abscesses.

**Conclusion:**

Advanced laboratory diagnostic techniques have improved the isolation and identification of nontuberculous mycobacteria. The diagnosis requires a high index of clinical suspicion, as detection by conventional methods is difficult. To our knowledge, this patient is the first documented case of cutaneous infection from this pathogen in Italy.

## Introduction

Infections due to environmental mycobacteria are emerging diseases not only in immunocompromised patients but also in immunocompetent hosts.

Because of the non-specific appearance of the skin lesions, dermatological infections from environmental mycobacteria are commonly misdiagnosed, making treatment of such conditions difficult and untimely [1]. In recent years, new species of environmental mycobacteria have emerged, associated with skin infections. This may also be due to the development of better identification techniques that can distinguish species previously considered to be identical.

Mycobacterium gordonae *(M. gordonae) *is an acid-and alcohol-fast bacillus belonging to the II group of scotochromogens mycobacteria. It is ubiquitous and commonly isolated from water (in fact, it was previously known as the "tap water bacillus" or *Mycobacterium aquae)*, soil and non-pasteurized fresh milk [1]. Nosocomial pseudo-infections due to antimicrobial and laboratory solutions, medical instrumentation, aerosol devices and continuous ambulatory peritoneal dialysis fluid have been reported [2]. The contamination of instrumentation is related to the high resistance of mycobacteria, and especially of *M. gordonae*, to common chemical disinfection [3], and to the practice of rinsing medical instruments with tap water after immersion in disinfectant [4]. Although *M.gordonae *is considered one of the least pathogenic among environmental mycobacteria, some cases of infections have occasionally been reported in immunocompromised and in HIV positive patients [1]. The skin and soft tissues, cornea, synovial tissue, meninges, prosthetic heart value, liver and peritoneum, and lower respiratory tract are most commonly involved [5]. The infection may also be disseminated [6]. We report on a case of *M. gordonae *infection in an 86-year-old woman and discuss the problems inherent to the identification and treatment of this emerging pathogen. To our knowledge, only few cases of cutaneous infection by *M. gordonae *have been described in literature, mostly occurring in immunocompetent patients [7]-[13].

## Case presentation

An 86-year-old Caucasian Italian woman was admitted to hospital for 6-months histories of a subcutaneous nodular lesion on the left leg. During hospitalization, a similar lesion appeared on the right clavicular region. Physical examination disclosed two slightly painful, fluctuant nodules with normal overlying skin, cool to the touch, mobile and measuring about 7 cm and 4 cm in width, respectively. There was no regional lymphadenopathy and the patient was afebrile. Routine laboratory tests did not show noteworthy alterations except for iron deficiency anemia (HGB = 10.1 g/dl, HCT = 31.0%). Chest X-ray was normal. Sputum and urine cultures were negative. Immunological evaluation including peripheral blood immunophenotyping, serum immunoglobulin levels and HC50 resulted normal. Anti-HIV antibodies were absent. Surgical drainage of both subcutaneous lesions was performed, evacuating yellowish pus of low viscosity. Direct microscopic examination of the smears from the two lesions yielded negative results for acid-fast bacilli, while cultures on Lowenstein-Jensen and Middlebrook 7H10 were positive 3 weeks later for mycobacteria. Intradermal tests with PPD of *Mycobacterium tuberculosis*, *Mycobacterium scrofulaceum *and *Mycobacterium avium *were positive, whereas tests for *Mycobacterium fortuitum, Mycobacterium marinum *and *Mycobacterium kansasii *were negative. A biopsy specimen obtained from the lesion on the left leg showed dermal granulomatous inflammation with caseous necrosis. Treatment with trimethoprim/sulphamethoxazole was started but after two months no result had been obtained. The patient was then re-hospitalized and underwent surgical drainage of the abscesses and a second culture from the smear. Contemporaneously, we obtained biochemical identification of the mycobacterium strain isolated from the abscesses as *Mycobacterium gordonae *(whose isolation was later confirmed in the second culture), diagnosis confirmed by microarray assay (LCD-Array Myco) DNA based identification of *Mycobacterium tuberculosis *complex (MTUB) and other Mycobacteria (MOT) [13,14]. In accordance with the results of the drug sensitivity test (Table 1), oral ciprofloxacin (1 g daily) was given. The skin lesions showed rapid improvement and resolved completely after 6 months of treatment. At the time of writing, 7 months after completing the treatment, there has been no evidence of recurrence.

**Table 1:** Biochemical identification and susceptibility to antibiotics of isolated *Mycobacterium gordonae*

Biochemical test	Result
Growth rate	37°C
Pigmentation in the dark	+
Niacin	-
Nitrate reduction	-
Semi-quantitative catalase (>45 mm)	+
68°C pH7 catalase	+
Urease	-
Iron uptake	-
Tween 80 hydrolysis	+
Mac-Conkey agar	-
- Ciprofloxacin	+
- Mynocicline	+
- Doxycicline	+
- Rifampicin	+
- Trimethopin/Sulfa and Kanamycin	-

## Conclusion

Skin infection can follow an episode of trauma even if the entry portal of the pathogen may appear slight or invisible. Clinical aspects of skin lesions range from nodules to abscesses and ulcers. The present case occurred in an immunocompetent woman with "cold abscess" and no obvious entry portal. The abscess, which appeared later on the clavicular region was probably related to autoinoculation, since bacteraemia seems an unlikely explanation [8].

The species isolated from both the abscesses was undoubtedly pathogenic since all the specific criteria for an infection due to *M. gordonae *were fulfilled [6].

The case we report is the first documented case of cutaneous infection from *M. gordonae *in Italy. The diagnosis requires a high index of clinical suspicion, as detection by conventional methods is difficult. *M. gordonae *is one of the least pathogenic of the mycobacteria. It is usually a contaminant or colonizer in patients who are not infected with HIV [1]. However, in patients who are infected with HIV and have severe immunosuppression (count of <100 CD4^+ ^cells/μL), *M. gordonae*. may infect the lungs, blood, bone marrow, and other organs [6,13]. Most cases of presumed infection have occurred in patients with trauma, underlying immunosuppression, or a prosthetic device [13]. Nevertheless, it is important to establish a timely diagnosis so that optimal treatment can be administered [13,14]. In fact, this organism is resistant to conventional antitubercular agents such as isoniazid and p-aminosalicylic acid [5]. Problems which may be caused by episodes of pseudoinfection include delay of appropriate therapy, overgrowth of cultures with contaminants, and risks and costs associated with further diagnostic procedures and unnecessary therapy (2, 4, 15). The clinical laboratory must expend time and resources to identify the samples isolates, determine their source, and notify physicians of the problem. Pseudoinfection with mycobacteria may be particularly troublesome because of the long periods needed for growth and identification of isolates.

*M. gordonae *strain we isolated was resistant to trimethoprim-sulfamethoxazole but sensitive to ciprofloxacin, and long term administration (six months) induced complete healing of the cutaneous abscesses.

## Abbreviations

*M. gordonae*: *Mycobacterium gordonae*.

## Consent

Written informed consent was obtained from the patient for publication of this case report and all accompanying images. A copy of the written consent is available for review by the Editor-in-Chief of this journal.

## Competing interests

The authors declare that they have no competing interests.

## Authors' contributions

FC and SV conceived the study and participated in patient management, acquisition of data, interpretation of data, and were major contributors in writing the manuscript. RC, DPG and TA participated in patient management, acquisition of data, and drafting of the manuscript. SC, CS, GFR and BA participated in reference research. BD and CA revised critically the manuscript adding substantial intellectual content. DVD and AG coordinated the study and patient management and revised critically the manuscript. All authors have read and approved the final manuscript.
